# Supplementary material for: Using cloud-based mobile technology for assessment of competencies among medical students
Source: PeerJ. 2013 Sep 17;1:e164. doi: 10.7717/peerj.164 (PMC3792179; doi:10.7717/peerj.164)
Supplement: Supplemental Information 1 [file peerj-01-164-s001.pdf]

Gmail by Google

David Solomon <dsolomonmsuedu@gmail.com>

# Determination if a study involves human subjects

**David Solomon** <dsolomon@msu.edu> Fri, Jan 13, 2012 at 7:31 PM
To: irb@msu.edu
Cc: Gary Ferenchick <Gary.Ferenchick@hc.msu.edu>

Dear Sir or Madam,

Gary Ferenchick, MD and I are planning a study titled " Establishing the reliability of an electronic clinical evaluation tool" and would like to request your determination of whether or not this study involves human subjects. If you agree this study does not involve human subjects, we would appreciate your documenting this in writing for our study records.

The study would use evaluation data collected by the College of Human Medicine on students participating in the Internal Medicine Basic Clerkship collected as a normal part of the students' educational program. Some of this data will be collected by the clerkship via an electronic evaluation tool that is the focus of this investigation. Other data will include other performance assessments collected as part of the students' other educational activities in their medical school curriculum. This second set of data are maintained by Ann Taft in the Office of Medical Educational Research and Development. Dr. Ferenchick maintains the clerkship data that we are planing on using in the study. He will give these data to Ms. Taft who will act as an Honest Broker and merge the two sets of data via the students' PID. She will then return to Dr Ferenchick and me a combined data set that will be stripped of identifiers. The data set will include several hundred students. We do not believe it would be possible to uniquely identify an individual student through any combination of the variables included in the data set.

We believe that since this data set will be compiled and de-identified by an honest broker who will not participate in the research study, these data do not constitute human subjects data and as such this investigation does not require oversight by the Michigan State University Human Research Program.

Again, if you agree, we would appreciate your acknowledging your agreement in writing for our study records. If you have questions or concerns about our protocol or feel these data would constitute human subject data, please let us know.

Thank you in advance for your help in this matter.

--
David J Solomon, PhD
A-202 E Fee Hall
MSU
E. Lansing, MI 48823 USA
+1 517 353-2037 Ext 223
dsolomonmsuedu@gmail.com

# Determination if a study involves human subjects

**IRB** <IRB@ora.msu.edu> Mon, Jan 23, 2012 at 3:44 PM
To: David Solomon <dsolomon@msu.edu>

Hi Dr. Solomon -

I asked the Chair to please "weight-in" and he believes that using the data as you describe, would not involve human subjects because:

1. The data are not collected for research purposes and someone (the honest broker) who has the authority to access identifiable data will create a de-identified data set that will be analyzed.
2. If these data are analyzed for research such analyses, this does not involve human subjects.

So you should be all set and this project will not need IRB review.

Have a great day -

*Sally*

Sally Conley
IRB Administrator I / Compliance Analyst
Direct Line: 884-0296

---

**From:** dsolomonmsuedu@gmail.com [mailto:dsolomonmsuedu@gmail.com] **On Behalf Of** David Solomon
**Sent:** Tuesday, January 17, 2012 3:54 PM
**To:** IRB
**Cc:** Gary Ferenchick; Brian Mavis
**Subject:** Re: Determination if a study involves human subjects

[Quoted text hidden]

# Determination if a study involves human subjects

**David Solomon** <dsolomon@msu.edu> Tue, Mar 13, 2012 at 3:19 PM
To: mcgeeh@ora.msu.edu
Cc: Brian Mavis <Brian.Mavis@hc.msu.edu>, Gary Ferenchick <Gary.Ferenchick@hc.msu.edu>

Hi Dr. Mcgee,

As I think you know, we have started using an "honest broker" in CHM to provide us with de-identified student performance data for research purposes to avoid human subject protection issues. I worked through getting a determination that this was appropriate and your office concurred as noted by Sally Conley below.

I just want to verify that as long as a project meets the criteria spelled out by Sally below we will not need to contact your office for approval of each new project. Sorry to bother you, but I just want to make sure we are all on the same page on this issue.

Thanks,

Dave Solomon

---------- Forwarded message ----------
From: **IRB** <IRB@ora.msu.edu>
Date: Mon, Jan 23, 2012 at 8:44 AM
[Quoted text hidden]
[Quoted text hidden]

965 Fee Road
MSU
E. Lansing, MI 48823 USA
+1 517 353-2037 Ext 223
dsolomon@msu.edu

Gmail by Google

David Solomon <dsolomonmsuedu@gmail.com>

# Determination if a study involves human subjects

**McGee, Harry** <mcgeeh@ora.msu.edu> Tue, Mar 13, 2012 at 3:26 PM
To: David Solomon <dsolomon@msu.edu>
Cc: Brian Mavis <Brian.Mavis@hc.msu.edu>, Gary Ferenchick <Gary.Ferenchick@hc.msu.edu>

Dr. Solomon,

Data that are already being collected for educational purposes, de-identified by someone other than the researcher who has authority to access these data (an honest broker) and then analyzed for research is research that does not involve human subjects and does not need MSU IRB review.

Harry


Harry McGee, Chair SIRB

Michigan State University

205 Olds Hall

East Lansing, MI 48824

517-355-2180


"If we knew what we were doing it wouldn't be called research"  Albert Einstein

**From:** dsolomonmsuedu@gmail.com [mailto:dsolomonmsuedu@gmail.com] **On Behalf Of** David Solomon
**Sent:** Tuesday, March 13, 2012 9:20 AM
**To:** McGee, Harry
**Cc:** Brian Mavis; Gary Ferenchick
**Subject:** Fwd: Determination if a study involves human subjects

[Quoted text hidden]
