# Supplementary material for: Using cloud-based mobile technology for assessment of competencies among medical students
Source: PeerJ. 2013 Sep 17;1:e164. doi: 10.7717/peerj.164 (PMC3792179; doi:10.7717/peerj.164)
Supplement: Supplemental Information 2 [file peerj-01-164-s002.zip › PeerJ Article Data/Interrater reliability CEX/Total.pdf]

Inter-Rater Reliability Calculator

Number of person/objects rated	6
Harmonic mean number of raters per person/object rated	17.00
Mean rating†	0.46
Standard Deviation of the ratings†	0.21

†The mean and standard deviation are based on the averaged ratings for each person/object rated.

The reliability for a score based on 1 rater would be 0.69

Breakdown for the ratings of each person/object rated:

ID	Mean	SD	N
1	0.24	0.04	17
2	0.80	0.10	17
3	0.24	0.17	17
4	0.44	0.08	17
5	0.59	0.26	17
6	0.43	0.06	17

SD = Standard Deviation   N = Number of ratings

NOTE: This program has been extensively tested and I believe the statistics provided above are accurate. This program however is provided as is without warranties as to its accuracy or fitness for a particular pupose. Please understand that you are using it at your own risk.
